# Supplementary material for: A LETM2-Regulated PI3K-Akt Signaling Axis Reveals a Prognostic and Therapeutic Target in Pancreatic Cancer
Source: Cancers (Basel). 2022 Sep 28;14(19):4722. doi: 10.3390/cancers14194722 (PMC9564284; doi:10.3390/cancers14194722)
Supplement: Supplementary file 1 [file cancers-14-04722-s001.zip › Supplementary Materials Figures.pdf]

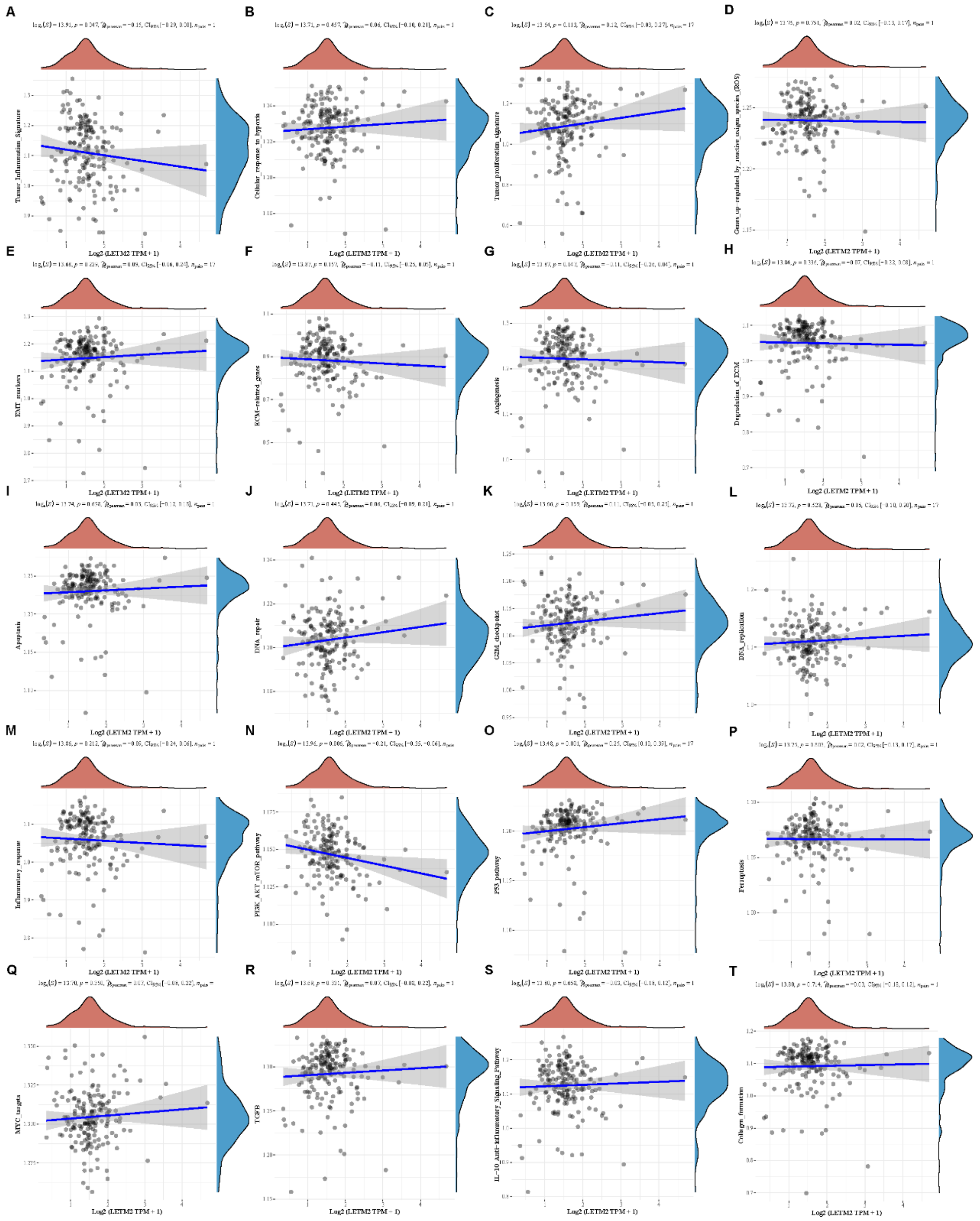

Figure S1. Correlation analysis between LETM2 expression and signaling pathway.

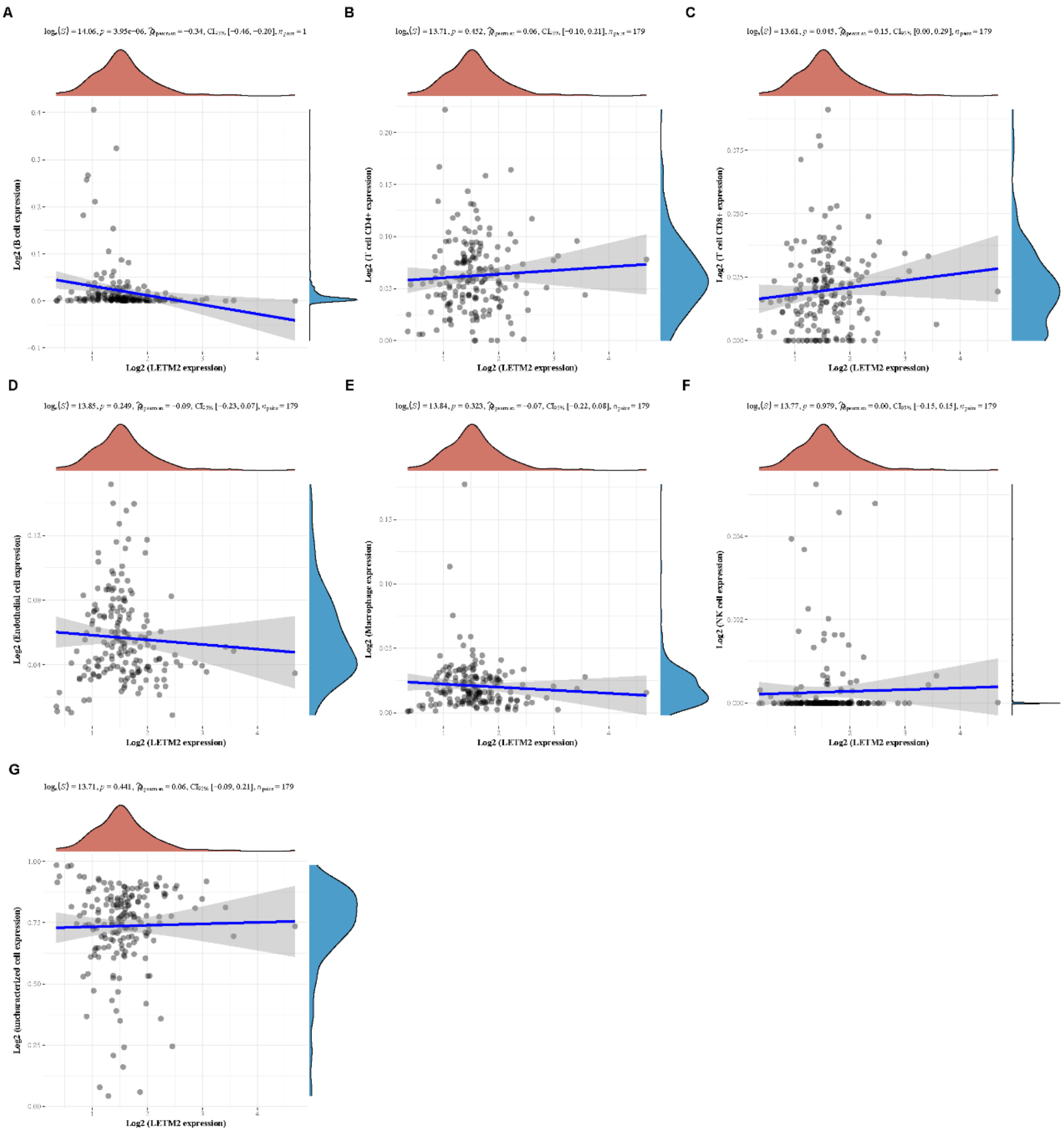

Figure S2. Correlation analysis between LETM2 expression and immune infiltration.

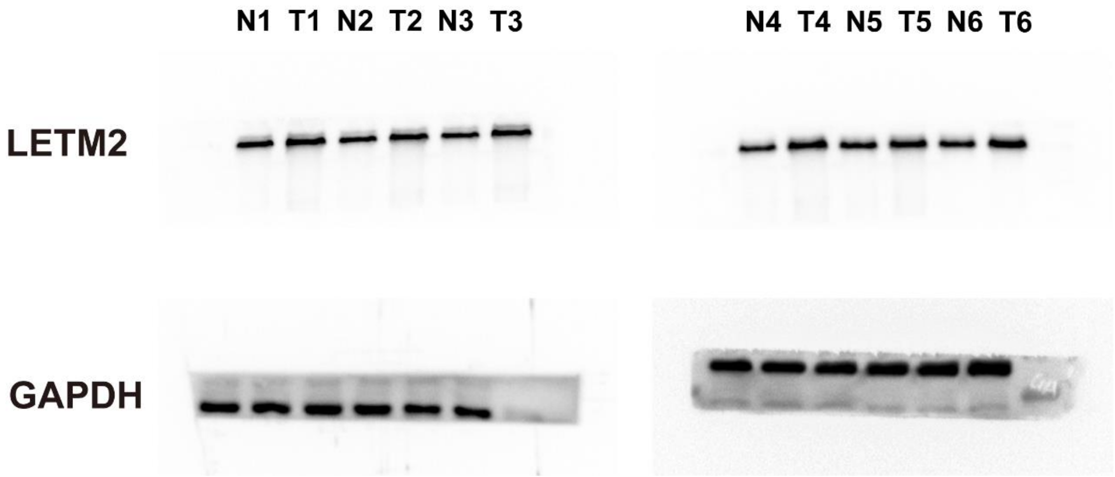

Figure S3. Source Western-blot Images for Figure 3A.

The quantification analysis for the western-blot could be found in Table S7.

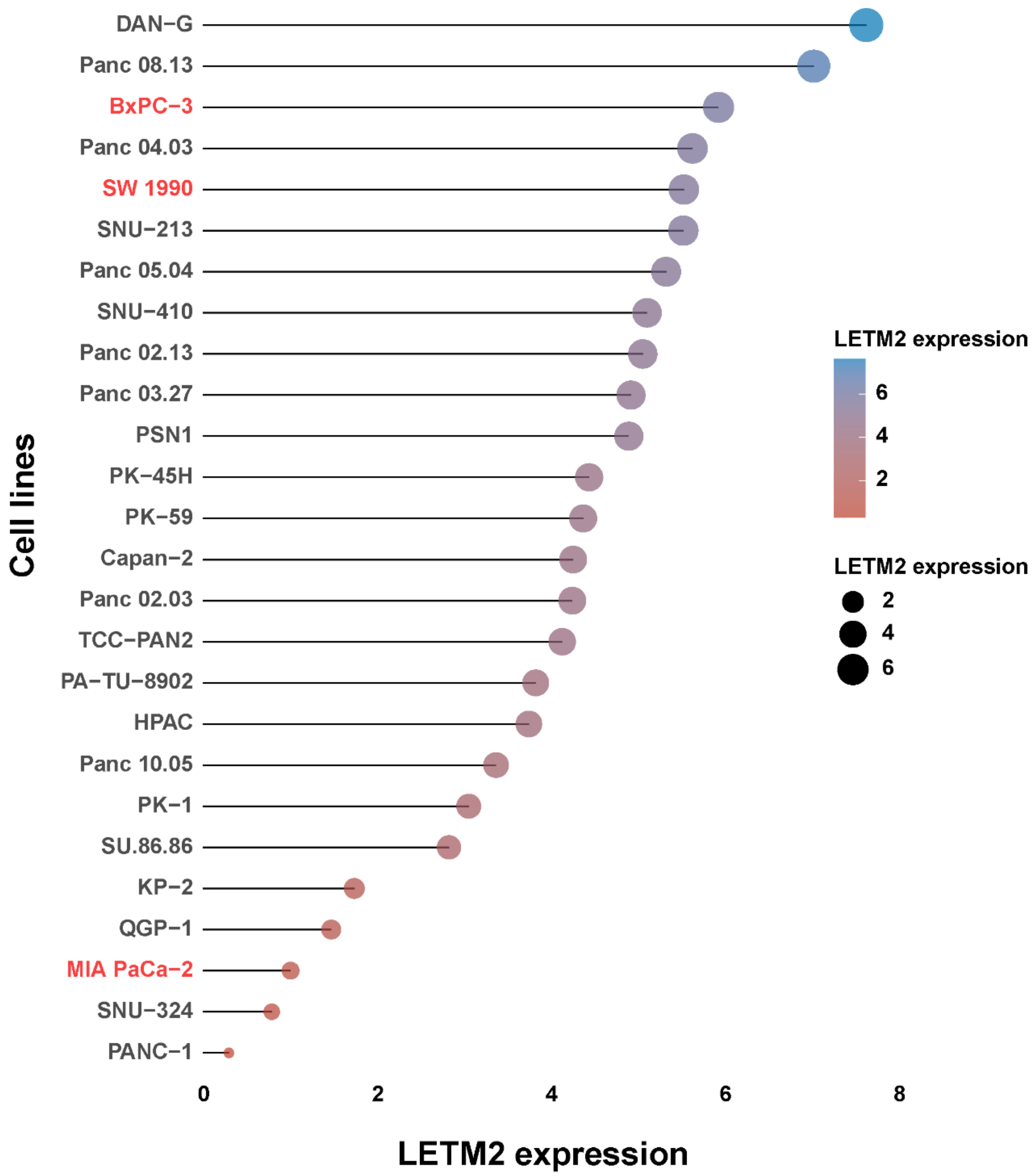

Figure S4. The bioinformatic analysis of relative expression of LETM2 in PAAD cell lines via CCLE database.

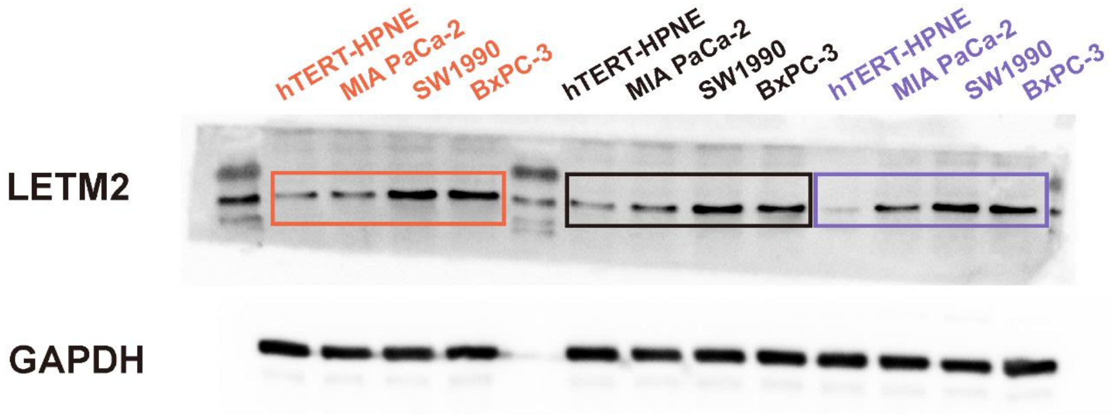

Figure S5. Source Western-blot Images for Figure 4A.

The orange, black, and purple frame indicates three independent experiments. The quantification analysis for the western-blot could be found in Table S9.

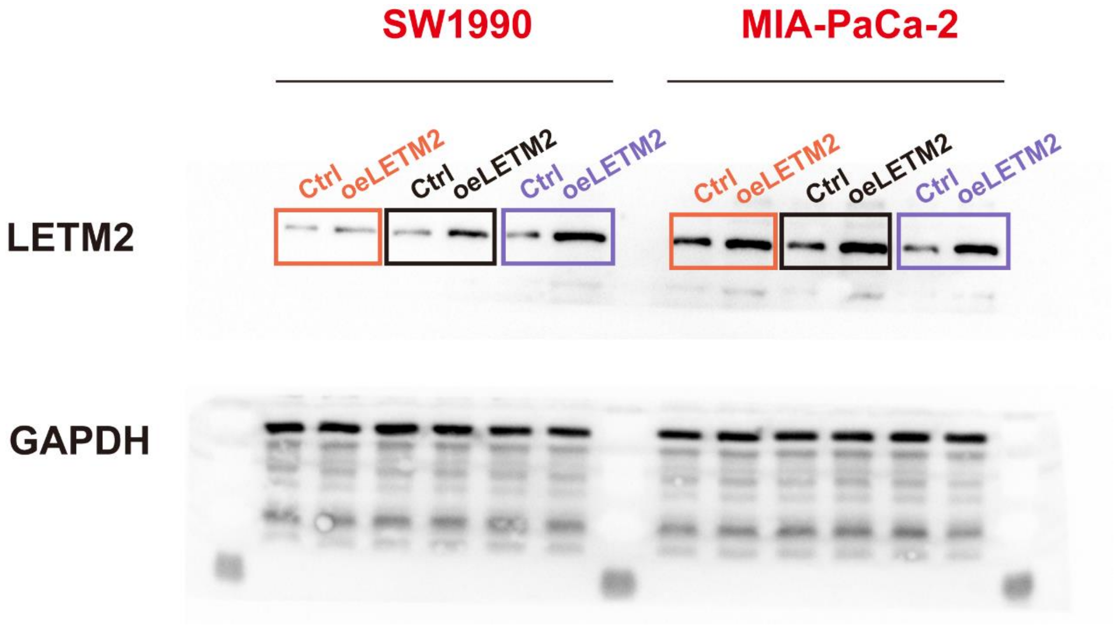

Figure S6. Source Western-blot Images for Figure 4D.

The orange, black, and purple frame indicates three independent experiments. The quantification analysis for the western-blot could be found in Table S10.

BxPc-3

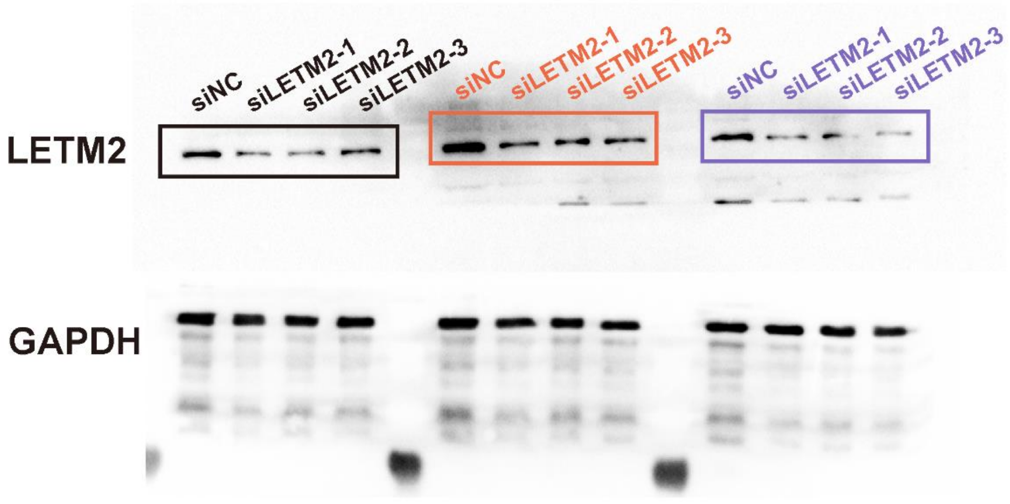

SW1990

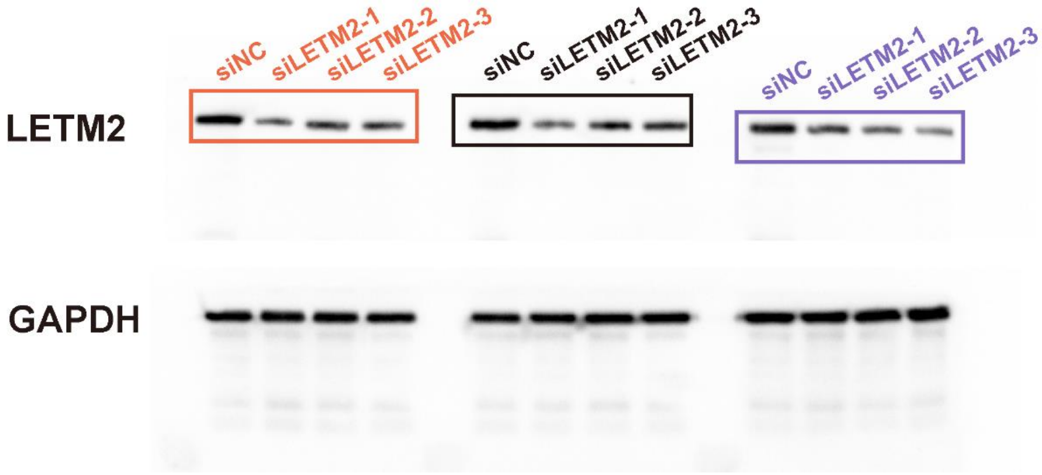

Figure S7. Source Western-blot Images for Figure 5A.

The orange, black, and purple frame indicates three independent experiments. The quantification analysis for the western-blot could be found in Table S11.

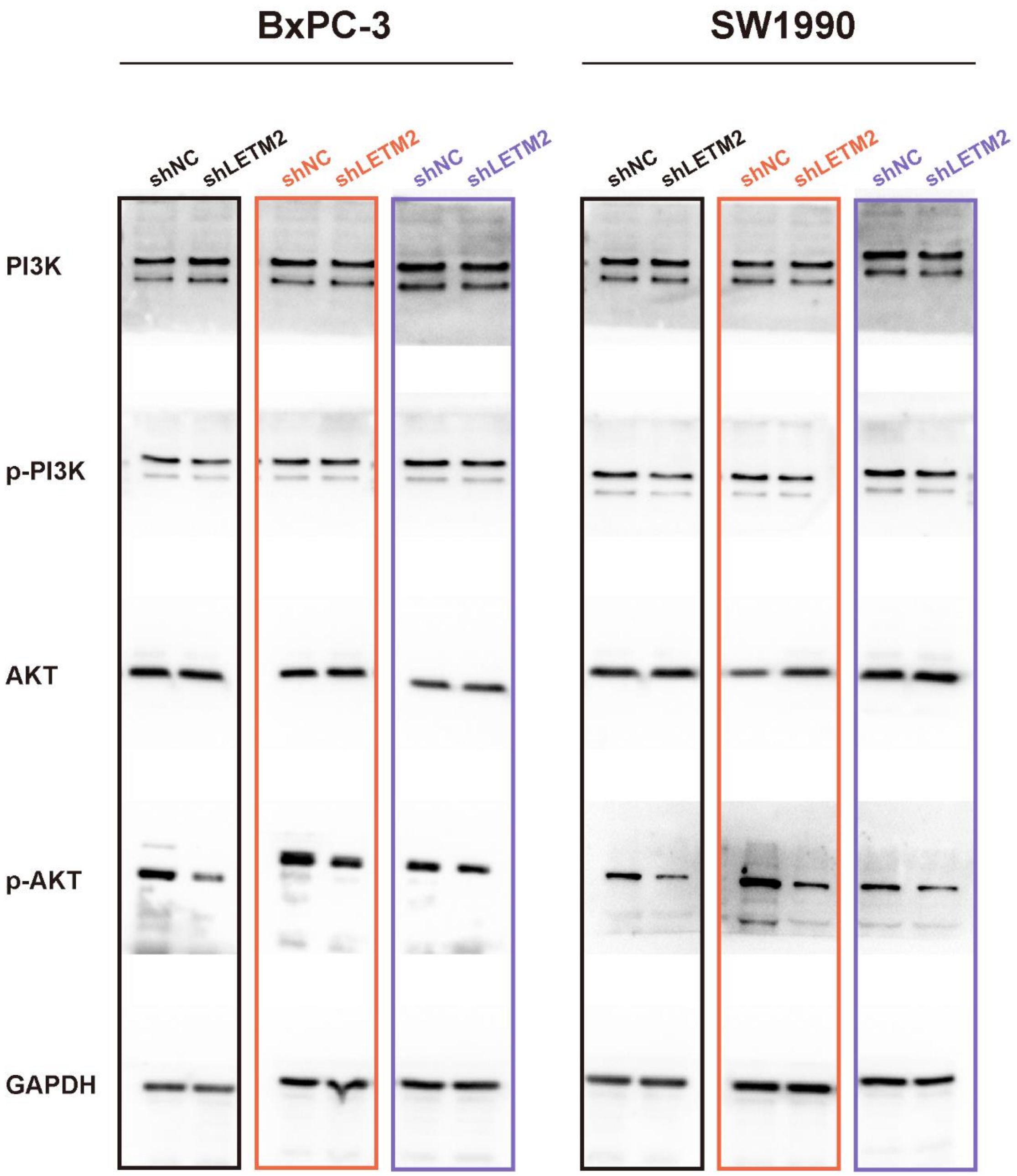

Figure S8. Source Western-blot Images for Figure 6E.

The orange, black, and purple frame indicates three independent experiments. The quantification analysis for the western-blot could be found in Table S12.
