# Supplementary material for: A LETM2-Regulated PI3K-Akt Signaling Axis Reveals a Prognostic and Therapeutic Target in Pancreatic Cancer
Source: Cancers (Basel). 2022 Sep 28;14(19):4722. doi: 10.3390/cancers14194722 (PMC9564284; doi:10.3390/cancers14194722)
Supplement: Supplementary file 1 [file cancers-14-04722-s001.zip › Table S10 Source data for Figure 4D.pdf]

Table S10 | Source data for Figure

4D SW1990

Target	Sample	Integrated Gray Density 1	Integrated Gray Density 2	Integrated Gray Density 3	Relative Expression 1	Relative Expression 2	Relative Expression 3
**LETM2**	Ctrl	32863	28660	19061	0.52	0.40	0.34
	oeLETM2	58207	76517	69319	0.91	1.18	1.33
**GAPDH**	Ctrl	63498	71844	56066			
	oeLETM2	63616	65110	52102			

MIA-PaCa-3

Target	Sample	Integrated Gray Density 1	Integrated Gray Density 2	Integrated Gray Density 3	Relative Expression 1	Relative Expression 2	Relative Expression 3
**LETM2**	Ctrl	33447	38189	24695	0.60	0.65	0.41
	oeLETM2	68856	79467	63610	1.10	1.38	1.12
**GAPDH**	Ctrl	56195	58401	60130			
	oeLETM2	62438	57501	56684			
