# Supplementary material for: A LETM2-Regulated PI3K-Akt Signaling Axis Reveals a Prognostic and Therapeutic Target in Pancreatic Cancer
Source: Cancers (Basel). 2022 Sep 28;14(19):4722. doi: 10.3390/cancers14194722 (PMC9564284; doi:10.3390/cancers14194722)
Supplement: Supplementary file 1 [file cancers-14-04722-s001.zip › Table S11 Source data for Figure 5A.pdf]

Table S11 | Source data for Figure

5A BxPC-2

Target	Sample	Integrated Gray Density 1	Integrated Gray Density 2	Integrated Gray Density 3	Relative Expression 1	Relative Expression 2	Relative Expression 3
**LETM2**	siNC	52230	61720	55441	0.87	1.06	0.89
	siLETM2-1	12142	24011	13293	0.25	0.46	0.23
	siLETM2-2	11623	26692	17537	0.23	0.54	0.33
	siLETM2-3	26745	25500	10378	0.44	0.47	0.23
**GAPDH**	siNC	60165	58437	62500			
	siLETM2-1	49342	52512	57301			
	siLETM2-2	50553	49282	53909			
	siLETM2-3	60147	54791	44369			

SW1990

Target	Sample	Integrated Gray Density 1	Integrated Gray Density 2	Integrated Gray Density 3	Relative Expression 1	Relative Expression 2	Relative Expression 3
**LETM2**	siNC	68293	74591	63702	0.87	0.99	0.68
	siLETM2-1	21502	26902	33087	0.29	0.33	0.37
	siLETM2-2	37601	39452	25201	0.49	0.44	0.27
	siLETM2-3	32038	41547	16779	0.42	0.47	0.18
**GAPDH**	siNC	78904	75377	93892			
	siLETM2-1	75240	80544	90594			
	siLETM2-2	76569	89702	93234			
	siLETM2-3	77095	87819	94174			
