# Supplementary material for: A LETM2-Regulated PI3K-Akt Signaling Axis Reveals a Prognostic and Therapeutic Target in Pancreatic Cancer
Source: Cancers (Basel). 2022 Sep 28;14(19):4722. doi: 10.3390/cancers14194722 (PMC9564284; doi:10.3390/cancers14194722)
Supplement: Supplementary file 1 [file cancers-14-04722-s001.zip › Table S12 Source data for Figure 6E.pdf]

Table S12 | Source data for Figure

6E BxPC-2

Target	Sample	Integrated Gray Density 1	Integrated Gray Density 2	Integrated Gray Density 3	Relative Expression 1	Relative Expression 2	Relative Expression 3
**PI3K**	shNC	67624	61538	78564	0.97	0.82	1.32
	shLETM2	49773	66997	72139	0.63	0.91	1.33
**p-PI3K**	shNC	52420	66556	67624	0.75	0.88	1.13
	shLETM2	35319	36736	30773	0.45	0.50	0.575
**AKT**	shNC	65241	67927	76475	0.93	0.90	1.28
	shLETM2	66802	69254	83782	0.851	0.94	1.54
**p-AKT**	shNC	91847	70236	81357	1.32	0.93	1.36
	shLETM2	47862	38112	30989	0.61	0.52	0.57
**GAPDH**	shNC	69780	75379	59672			
	shLETM2	78420	73531	54387			

SW1990

Target	Sample	Integrated Gray Density 1	Integrated Gray Density 2	Integrated Gray Density 3	Relative Expression 1	Relative Expression 2	Relative Expression 3
**PI3K**	shNC	70888	61694	79352	1.18	0.80	1.12
	shLETM2	68365	61781	75002	1.04	0.88	1.05
**p-PI3K**	shNC	61310	58107	59949	1.02	0.76	0.84
	shLETM2	33320	21939	31343	0.51	0.31	0.44
**AKT**	shNC	62883	58409	73014	1.05	0.76	1.03
	shLETM2	64441	57206	80580	0.98	0.81	1.12
**p-AKT**	shNC	51800	60830	63745	0.86	0.79	0.90
	shLETM2	30510	26323	38171	0.46	0.37	0.53
**GAPDH**	shNC	60111	76830	71019			
	shLETM2	65908	70595	71713			
