# Supplementary material for: A LETM2-Regulated PI3K-Akt Signaling Axis Reveals a Prognostic and Therapeutic Target in Pancreatic Cancer
Source: Cancers (Basel). 2022 Sep 28;14(19):4722. doi: 10.3390/cancers14194722 (PMC9564284; doi:10.3390/cancers14194722)
Supplement: Supplementary file 1 [file cancers-14-04722-s001.zip › Table S2 Primers used in qRT-PCR analysis.pdf]

Table S2 | Primers used in qRT-PCR analysis

Target		Primer Sequences (5' - 3')
**LETM2**	Forward	GCCCTGGAACACTTAGATCG
	Reverse	TGTTGTCGCAGTTGTTCCTC
**GAPDH**	Forward	TCAACGGATTTGGTCGTATTGGGCG
	Reverse	CTCGCTCCTGGAAGATGGTGATGGG
