# Supplementary material for: A LETM2-Regulated PI3K-Akt Signaling Axis Reveals a Prognostic and Therapeutic Target in Pancreatic Cancer
Source: Cancers (Basel). 2022 Sep 28;14(19):4722. doi: 10.3390/cancers14194722 (PMC9564284; doi:10.3390/cancers14194722)
Supplement: Supplementary file 1 [file cancers-14-04722-s001.zip › Table S3 LETM2 siRNA sequences.pdf]

Table S3 | LETM2 siRNA sequences

Name		RNA Oligo Sequences (5' - 3')
**siLETM2-1**	sense	GGAACUAGACUAAUACAAAdTdT
	antisense	UUUGUAUUAGUCUAGUUCCdTdT
**siLETM2-2**	sense	GGCUGUAAAGUUGGAACUAdTdT
	antisense	UAGUUCCAACUUUACAGCCdTdT
**siLETM2-3**	sense	GGUGGAUCUUGCACCUCAAdTdT
	antisense	UUGAGGUGCAAGAUCCACCdTdT
