# Supplementary material for: A LETM2-Regulated PI3K-Akt Signaling Axis Reveals a Prognostic and Therapeutic Target in Pancreatic Cancer
Source: Cancers (Basel). 2022 Sep 28;14(19):4722. doi: 10.3390/cancers14194722 (PMC9564284; doi:10.3390/cancers14194722)
Supplement: Supplementary file 1 [file cancers-14-04722-s001.zip › Table S4 Pan-cancer analysis of LETM2 expression in TCGA dataset..pdf]

Table S4 | Pan-cancer analysis of LETM2 expression in TCGA dataset.

	Tumor	Normal	P value
**Upregulation**			
CESC	1.94±1.59	0.48±0.38	0.05
LUAD	1.79±1.43	1.27±0.75	5.7e-4
COAD	0.54±1.28	-0.85±0.45	7.0e-15
COADREAD	0.49±1.23	-0.76±0.53	1.4e-15
BRCA	1.33±1.40	0.50±0.66	5.2e-13
ESCA	1.97±1.38	0.81±1.52	5.8e-3
STES	1.55±1.36	0.69±0.96	5.3e-6
KIRP	2.49±1.11	2.21±0.86	3.0e-4
STAD	1.37±1.31	0.65±0.68	8.4e-4
HNSC	2.25±1.64	0.52±1.43	3.2e-10
LIHC	-1.38±1.78	-2.54±1.48	3.8e-7
READ	0.31±1.06	-0.38±0.66	0.03
PAAD	2.98±1.27	1.24±1.31	0.02
PCPG	2.68±0.78	-1.01±0.85	3.4e-3
CHOL	2.15±1.95	-2.25±0.94	6.4e-6
**Downregulation**			
KIPAN	1.83±1.14	2.21±0.86	8.5e-5
PRAD	-0.53±0.77	-0.02±0.53	5.5e-7
KIRC	1.50±1.02	2.21±0.86	2.2e-14
THCA	0.06±0.71	0.24±0.66	0.03
KICH	1.63±0.98	2.21±0.86	5.7e-5
