# Supplementary material for: A LETM2-Regulated PI3K-Akt Signaling Axis Reveals a Prognostic and Therapeutic Target in Pancreatic Cancer
Source: Cancers (Basel). 2022 Sep 28;14(19):4722. doi: 10.3390/cancers14194722 (PMC9564284; doi:10.3390/cancers14194722)
Supplement: Supplementary file 1 [file cancers-14-04722-s001.zip › Table S5 Pan-cancer analysis of LETM2 expression in TCGA+GTEx dataset.pdf]

Table S5 | Pan-cancer analysis of LETM2 expression in TCGA+GTEx dataset.

	Tumor	Normal	P value
**Upregulation**			
GBM	1.74±0.68	1.71±1.64	0.01
GBMLGG	1.74±0.63	1.71±1.64	7.7e-6
LGG	1.74±0.62	1.71±1.64	3.4e-5
BRCA	1.33±1.40	0.57±0.63	5.5e-24
LUAD	1.79±1.43	1.11±0.83	1.4e-13
ESCA	1.97±1.38	0.82±1.16	3.5e-23
STES	1.55±1.36	0.65±1.27	1.0e-30
KIRP	2.49±1.11	1.98±1.42	1.8e-6
STAD	1.37±1.31	0.12±1.44	1.7e-24
HNSC	2.25±1.64	0.52±1.43	3.2e-10
LIHC	-1.38±1.78	-2.22±1.51	4.1e-8
SKCM	0.65±0.90	-0.10±0.66	4.4e-17
READ	0.31±1.06	-0.38±0.66	0.03
PAAD	2.98±1.27	0.16±1.29	5.6e-53
ALL	1.19±1.11	0.37±1.28	1.2e-10
LAML	1.82±0.65	0.37±1.28	4.1e-38
PCPG	2.68±0.78	-1.01±0.85	3.4e-3
ACC	0.87±1.04	-0.78±1.27	2.9e-19
CHOL	2.15±1.95	-2.25±0.94	6.4e-6
**Downregulation**			
KIPAN	1.83±1.14	1.98±1.42	4.9e-3
PRAD	-0.53±0.77	0.51±0.69	3.4e-38
KIRC	1.50±1.02	1.98±1.42	2.6e-11
WT	1.30±0.67	1.98±1.42	1.7e-12
THCA	0.06±0.71	0.33±0.82	4.7e-11
TGCT	0.66±0.92	4.94±1.17	2.2e-48
KICH	1.63±0.98	1.98±1.42	1.8e-3
