# Supplementary material for: A LETM2-Regulated PI3K-Akt Signaling Axis Reveals a Prognostic and Therapeutic Target in Pancreatic Cancer
Source: Cancers (Basel). 2022 Sep 28;14(19):4722. doi: 10.3390/cancers14194722 (PMC9564284; doi:10.3390/cancers14194722)
Supplement: Supplementary file 1 [file cancers-14-04722-s001.zip › Table S6 Pan-cancer analysis of LETM2 expression in GEPIA database..pdf]

Table S6 | Pan-cancer analysis of LETM2 expression in GEPIA dataset.

Cancer Type	Tumor	Normal	P value
**ACC**	1.88	0.6	
**BLCA**	1.95	1.48	
**BRCA**	2.51	1.52	
**CESC**	3.13	2.48	
**CHOL**	5.42	0.21	＜0.05
**COAD**	1.3	1.41	
**DLBC**	1.64	1.34	
**ESCA**	3.47	1.32	
**GBM**	3.43	3.2	
**HNSC**	4.44	1.4	＜0.05
**KICH**	3.24	3.55	
**KIRC**	2.68	3.6	
**KIRP**	6.37	3.1	
**LAML**	3.52	2.27	
**LGG**	3.27	3.2	
**LIHC**	0.35	0.24	
**LUAD**	3.16	2.11	
**LUSC**	2.25	2.13	
**OV**	2.23	2.36	
**PDAC**	8.11	1.18	＜0.05
**PCPG**	6.66	0.48	＜0.05
**PRAD**	0.7	1.45	
**READ**	1.18	1.61	
**SARC**	2.93	1.52	
**SKCM**	1.63	0.93	
**STAD**	2.31	1.2	
**TGCT**	1.39	38.71	＜0.05
**THCA**	1.04	1.32	
**THYM**	1.95	1.35	
**UCEC**	3.65	2.15	
**UCS**	2.61	2.2	

The value represents the median expression of certain tumor type or normal tissue.
