# Supplementary material for: A LETM2-Regulated PI3K-Akt Signaling Axis Reveals a Prognostic and Therapeutic Target in Pancreatic Cancer
Source: Cancers (Basel). 2022 Sep 28;14(19):4722. doi: 10.3390/cancers14194722 (PMC9564284; doi:10.3390/cancers14194722)
Supplement: Supplementary file 1 [file cancers-14-04722-s001.zip › Table S7 Source data for Figure 3A.pdf]

Table S7 | Source data for Figure 3A

Target	Sample	Integrated Gray Density 1	Integrated Gray Density 2	Integrated Gray Density 3	Relative Expression 1	Relative Expression 2	Relative Expression 3
**LETM2**	Non-tumor	19687	19645	19427	0.65	0.63	0.80
	Tumor	27184	25643	27353	0.92	0.87	1.12
**GAPDH**	Non-tumor	30399	31305	24212			
	Tumor	29652	29470	24452			

Target	Sample	Integrated Gray Density 4	Integrated Gray Density 5	Integrated Gray Density 6	Relative Expression 4	Relative Expression 5	Relative Expression 6
**LETM2**	Non-tumor	26323	30641	30015	0.87	0.99	0.79
	Tumor	38415	36651	38758.5	1.30	1.15	0.99
**GAPDH**	Non-tumor	15146	15494	18959			
	Tumor	14721	15889	19489			
