# Supplementary material for: A LETM2-Regulated PI3K-Akt Signaling Axis Reveals a Prognostic and Therapeutic Target in Pancreatic Cancer
Source: Cancers (Basel). 2022 Sep 28;14(19):4722. doi: 10.3390/cancers14194722 (PMC9564284; doi:10.3390/cancers14194722)
Supplement: Supplementary file 1 [file cancers-14-04722-s001.zip › Table S8 The correlation between IHC score of LETM2 and the clinical characteristic in PDAC patients..pdf]

Table S8 | The correlation between IHC score of LETM2 and the clinical characteristic in PDAC patients.

Group	n	IHC score of LETM2, mean	Difference ± SEM	95% Confidence Interval	P value
**Total**	60				
**Gender**			0.4200 ± 0.6781	-0.9342, 1.774	0.5378
Female	25	6.080			
Male	35	6.500			
**Age**			-0.6251 ± 0.7018	-2.030, 0.7798	0.3768
≤65	29	6.690			
>65	31	6.065			
**T stage**			1.843 ± 0.8223	0.1969, 3.489	**0.0289**
T1+T2	13	4.923			
T3+T4	47	6.766			
**N stage**			1.273 ± 0.6898	-0.1081, 2.654	0.0702
N0	27	5.667			
N1-3	33	6.939			
**M stage**			1.733 ± 0.7831	0.1658, 3.301	**0.0308**
M0	44	5.933			
M1	16	7.667			
**Pathological stage**			1.514 ± 0.692	0.1270, 2.90	**0.0329**
I + II	24	5.458			
III + IV	36	6.972			
