# Supplementary material for: A LETM2-Regulated PI3K-Akt Signaling Axis Reveals a Prognostic and Therapeutic Target in Pancreatic Cancer
Source: Cancers (Basel). 2022 Sep 28;14(19):4722. doi: 10.3390/cancers14194722 (PMC9564284; doi:10.3390/cancers14194722)
Supplement: Supplementary file 1 [file cancers-14-04722-s001.zip › Table S9 Source data for Figure 4A.pdf]

## Table S9 | Source data for Figure 4A

| Target | Sample | Integrated Gray Density 1 | Integrated Gray Density 2 | Integrated Gray Density 3 | Relative Expression 1 | Relative Expression 2 | Relative Expression 3 |
|---|---|---|---|---|---|---|---|
| **LETM2** | hTERT-HPNE | 69905 | 69905 | 29952 | 0.91 | 0.90 | 0.41 |
| | MIA PaCa-2 | 95447 | 95447 | 123745 | 1.50 | 1.56 | 1.77 |
| | SW1990 | 221180 | 221180 | 199687 | 3.39 | 3.20 | 3.06 |
| | BxPC-3 | 216208 | 216208 | 193555 | 2.92 | 2.90 | 2.57 |
| **GAPDH** | hTERT-HPNE | 76732 | 78027 | 73870 | | | |
| | MIA PaCa-2 | 63491 | 61149 | 69840 | | | |
| | SW1990 | 65276 | 69040 | 65212 | | | |
| | BxPC-3 | 74021 | 74437 | 75210 | | | |
