# Supplementary figures and images for: A LETM2-Regulated PI3K-Akt Signaling Axis Reveals a Prognostic and Therapeutic Target in Pancreatic Cancer
Source: Cancers (Basel). 2022 Sep 28;14(19):4722. doi: 10.3390/cancers14194722 (PMC9564284; doi:10.3390/cancers14194722)

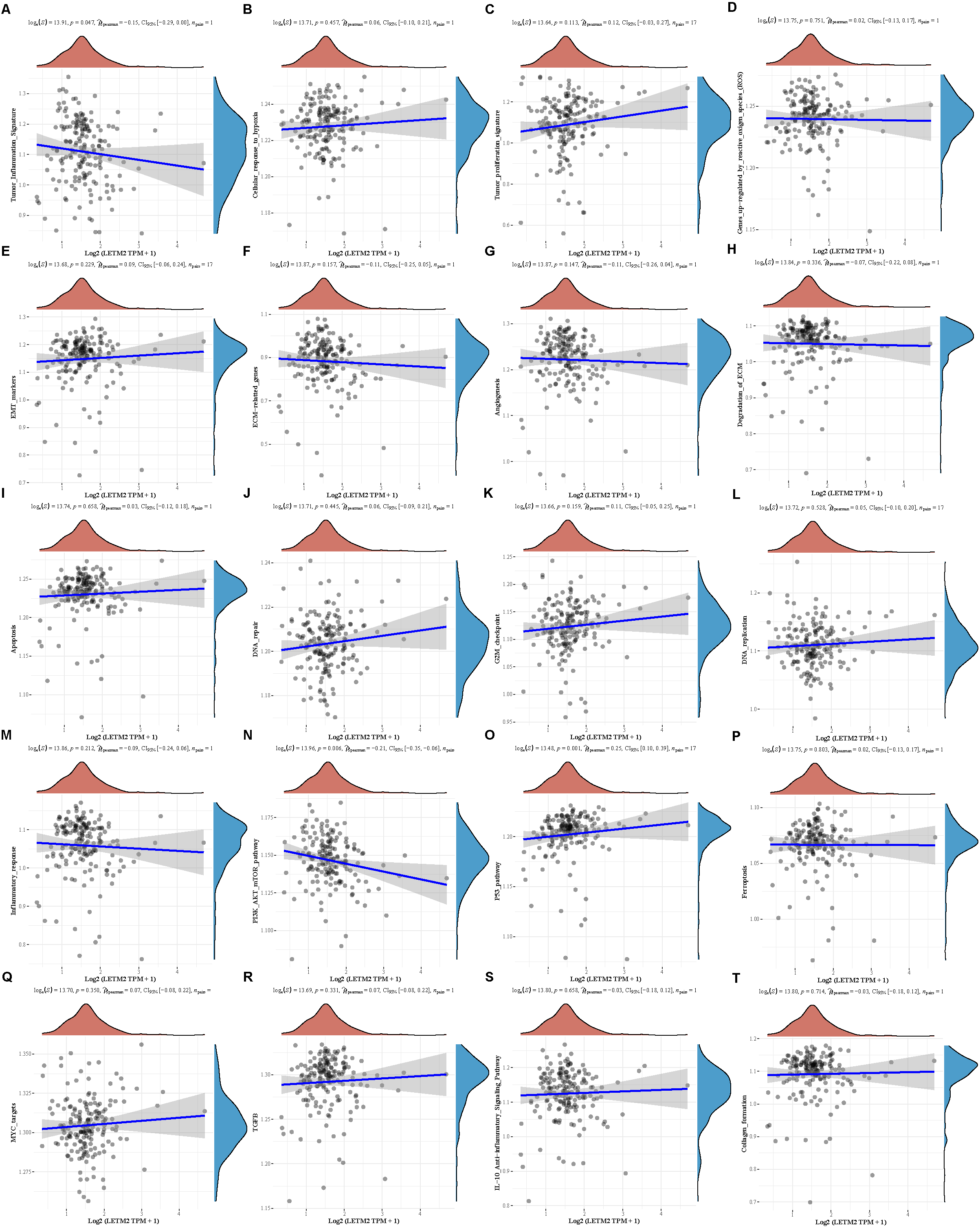

Supplement: Supplementary file 1 [file cancers-14-04722-s001.zip › Figure S1 Correlation analysis between LETM2 expression and signaling pathway..tif]

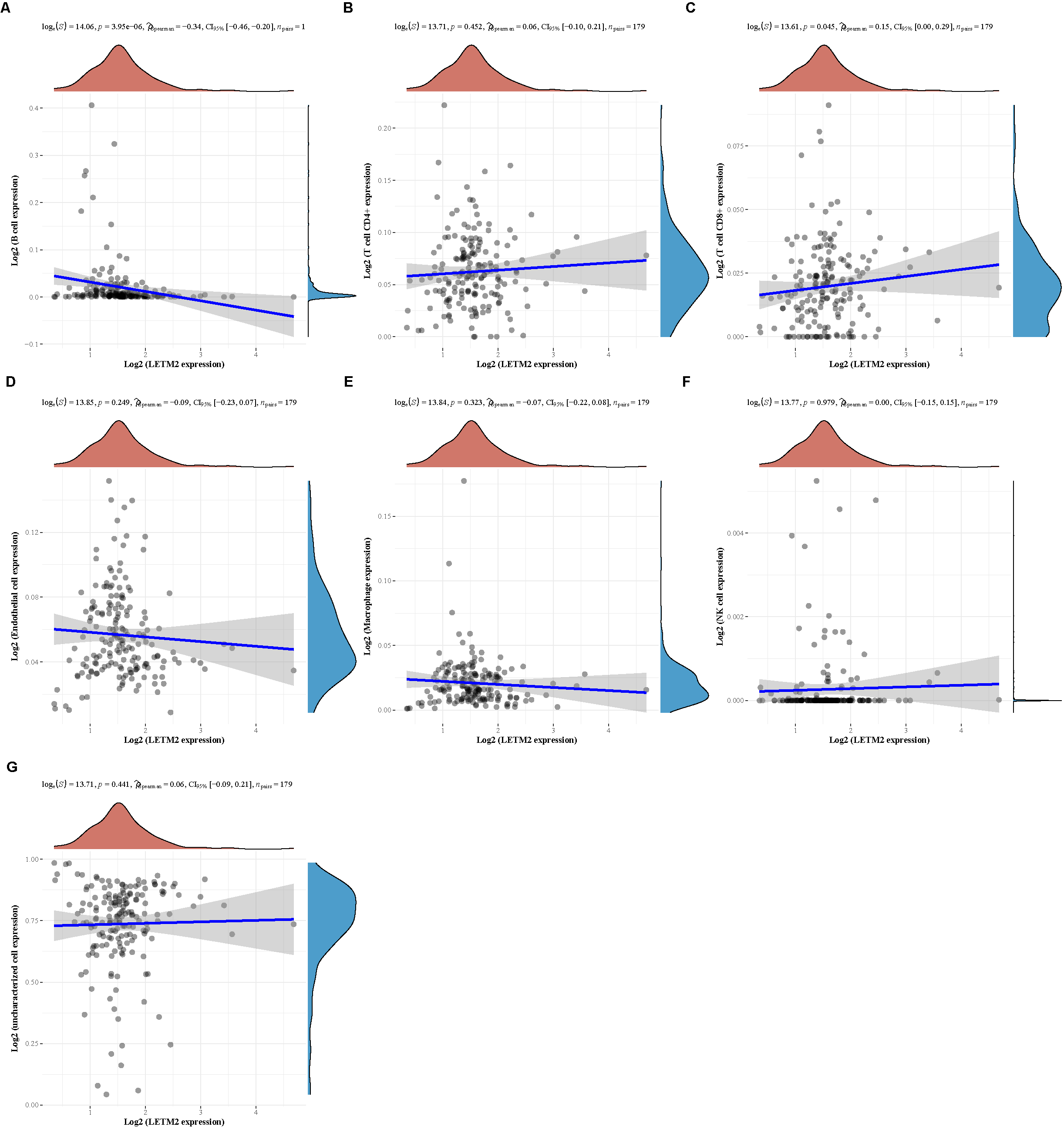

Supplement: Supplementary file 1 [file cancers-14-04722-s001.zip › Figure S2 Correlation analysis between LETM2 expression and immune infiltration..tif]

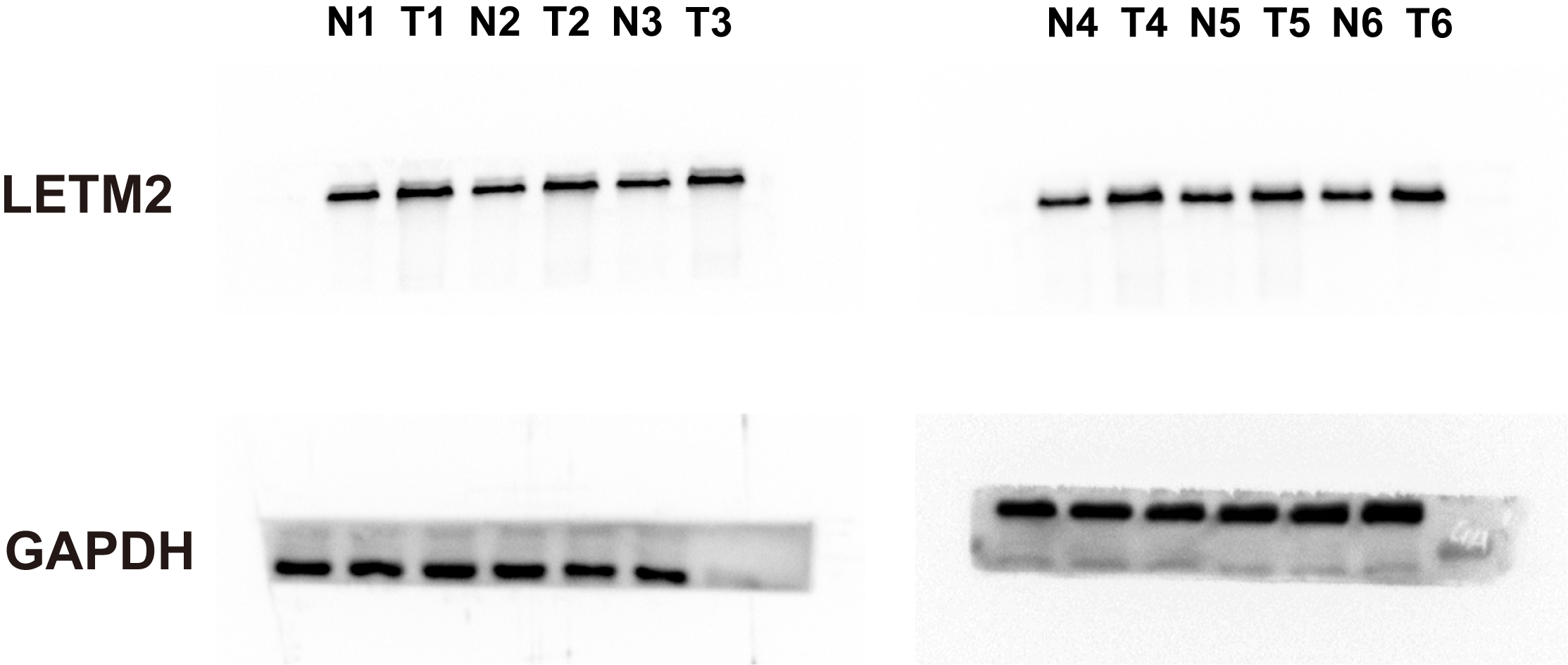

Supplement: Supplementary file 1 [file cancers-14-04722-s001.zip › Figure S3 Source Western-blot Images for Figure 3A.tif]

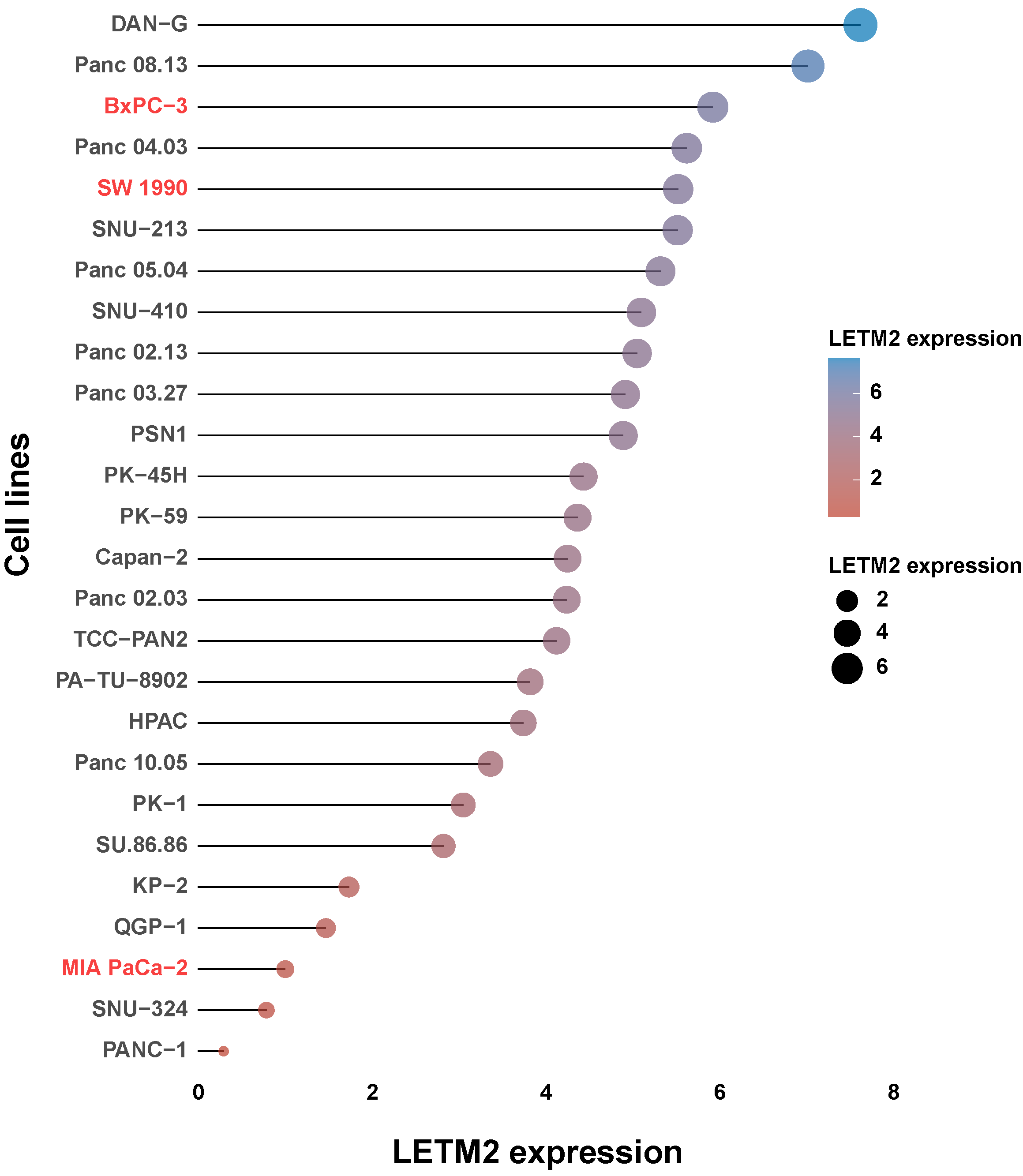

Supplement: Supplementary file 1 [file cancers-14-04722-s001.zip › Figure S4 The bioinformatic analysis of relative expression of LETM2 in PAAD cell lines via CCLE database.tif]

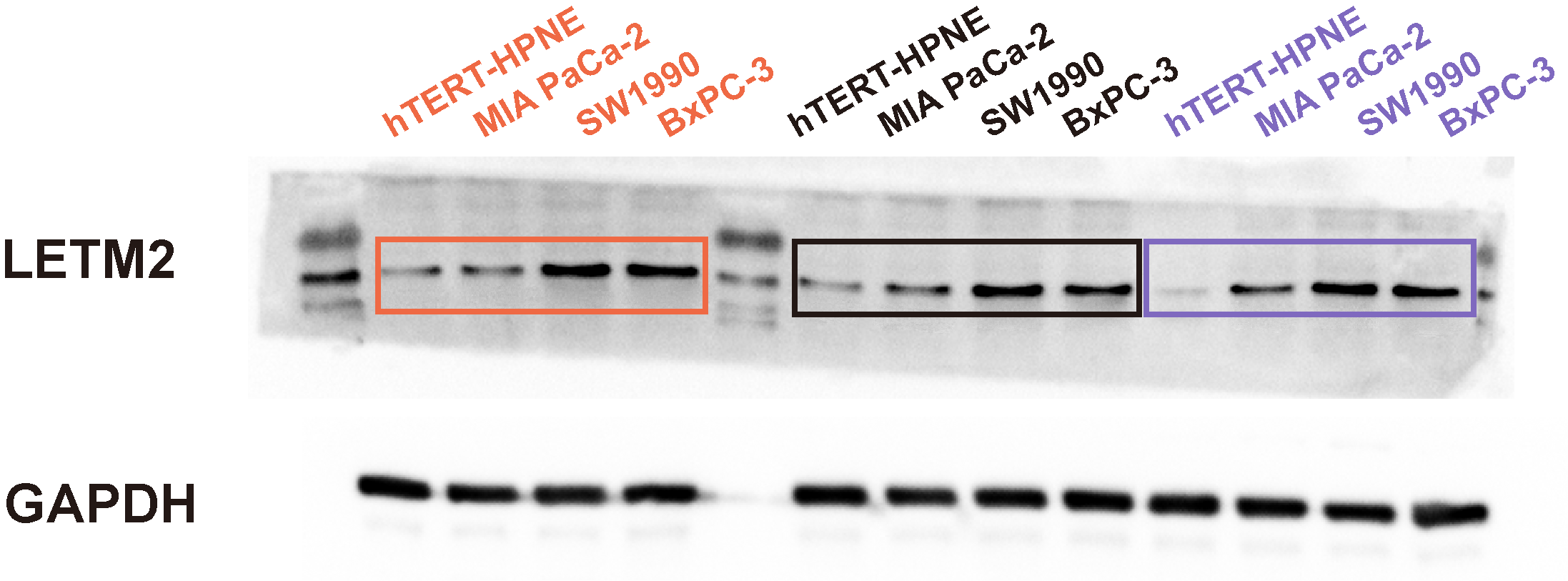

Supplement: Supplementary file 1 [file cancers-14-04722-s001.zip › Figure S5 Source Western-blot Images for Figure 4A.tif]

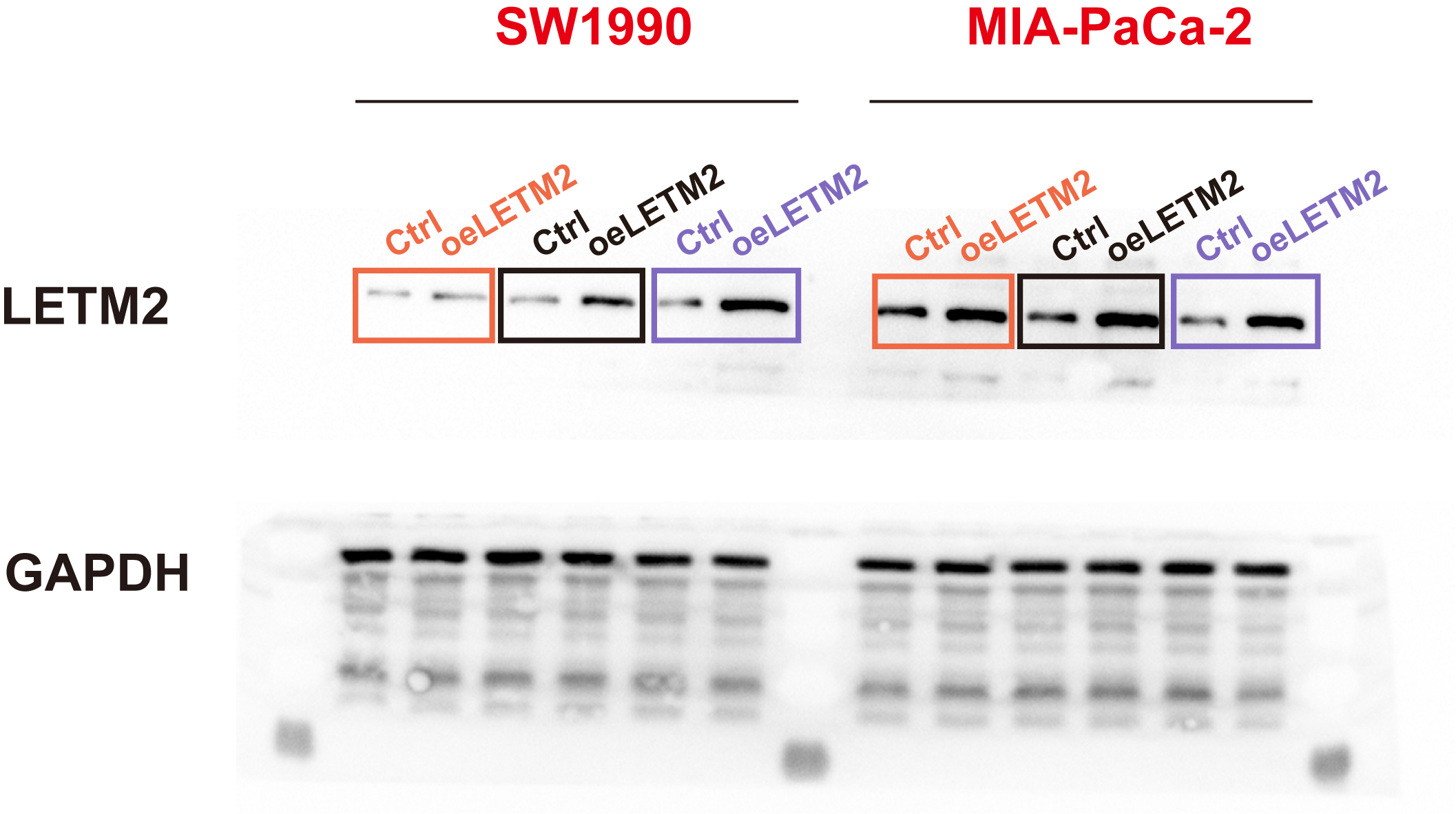

Supplement: Supplementary file 1 [file cancers-14-04722-s001.zip › Figure S6 Source Western-blot Images for Figure 4D.tif]

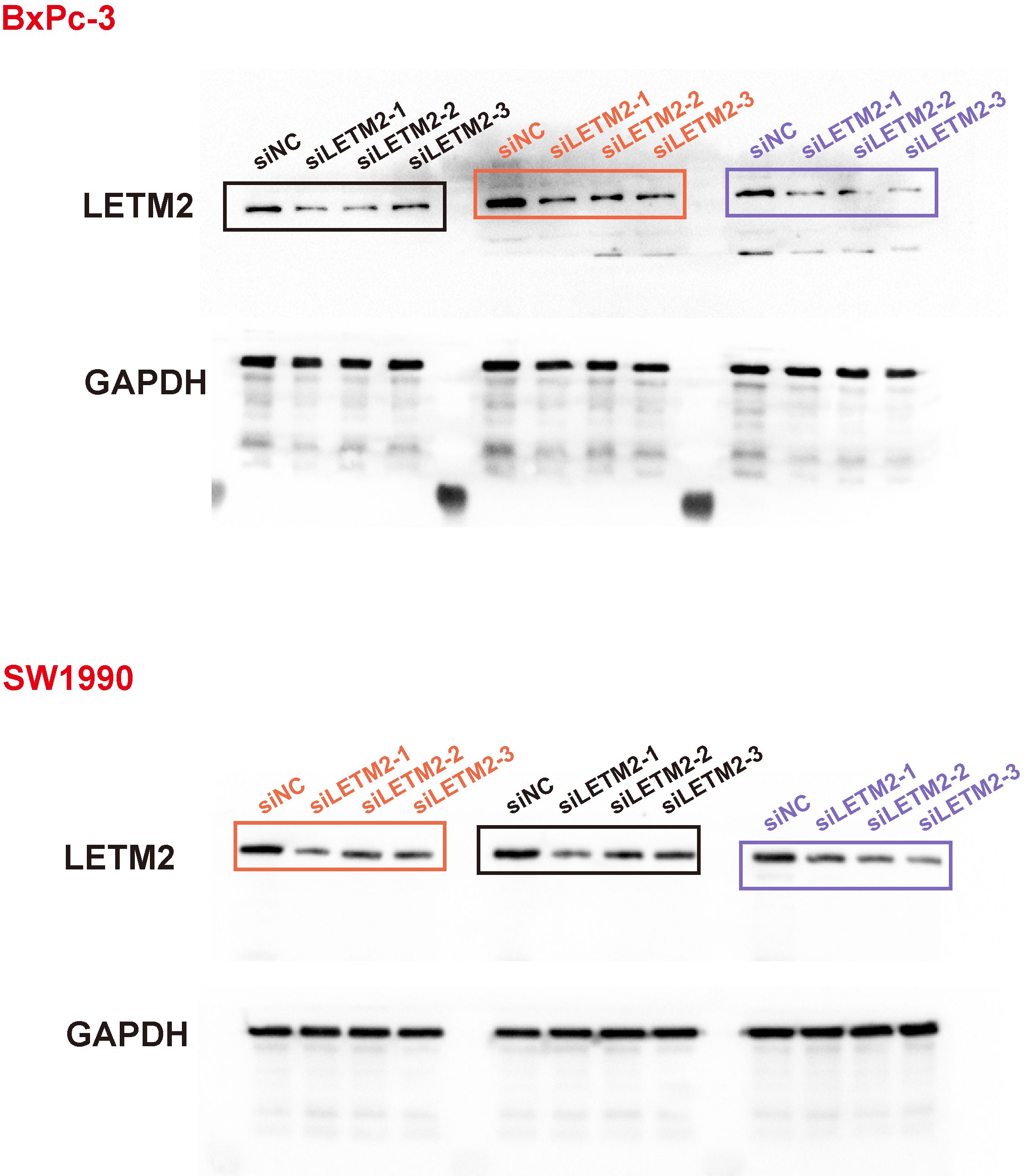

Supplement: Supplementary file 1 [file cancers-14-04722-s001.zip › Figure S7 Source Western-blot Images for Figure 5A.tif]

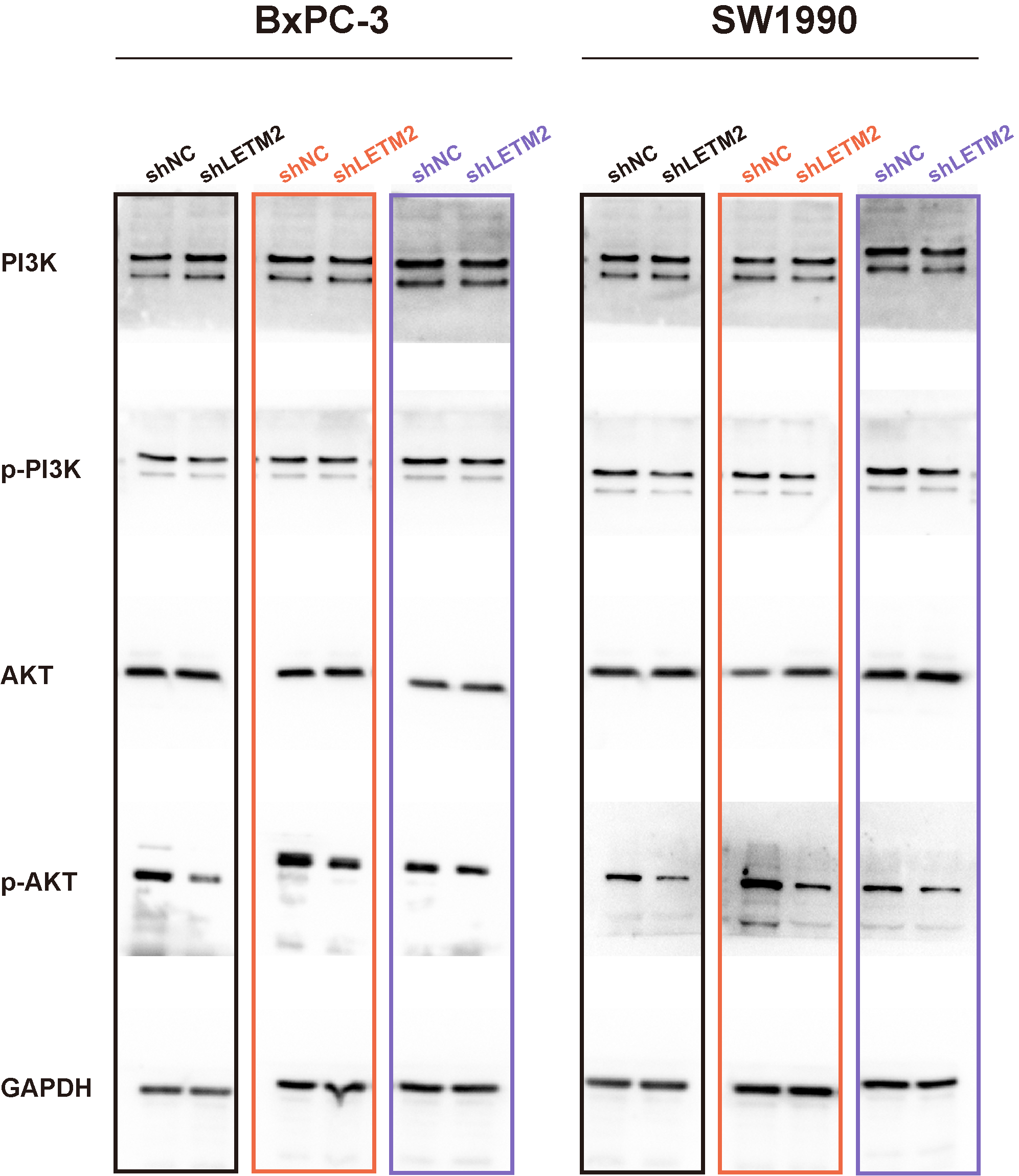

Supplement: Supplementary file 1 [file cancers-14-04722-s001.zip › Figure S8 Source Western-blot Images for Figure 6E.tif]
